# Review on the Use of Artificial Intelligence to Predict Fire Performance of Construction Materials and Their Flame Retardancy

**DOI:** 10.3390/molecules26041022

**Published:** 2021-02-15

**Authors:** Hoang T. Nguyen, Kate T. Q. Nguyen, Tu C. Le, Guomin Zhang

**Affiliations:** 1 Department of Infrastructure Engineering, School of Engineering, RMIT University, GPO Box 2476, Melbourne, VIC 3001, Australia; S3814773@student.rmit.edu.au (H.T.N.); kevin.zhang@rmit.edu.au (G.Z.); 2 Manufacturing, Materials and Mechatronics, School of Engineering, RMIT University, GPO Box 2476, Melbourne, VIC 3001, Australia; tu.le@rmit.edu.au

**Keywords:** flame retardants, combustion, chemical kinetics, pyrolysis, artificial intelligence, machine learning

## Abstract

The evaluation and interpretation of the behavior of construction materials under fire conditions have been complicated. Over the last few years, artificial intelligence (AI) has emerged as a reliable method to tackle this engineering problem. This review summarizes existing studies that applied AI to predict the fire performance of different construction materials (e.g., concrete, steel, timber, and composites). The prediction of the flame retardancy of some structural components such as beams, columns, slabs, and connections by utilizing AI-based models is also discussed. The end of this review offers insights on the advantages, existing challenges, and recommendations for the development of AI techniques used to evaluate the fire performance of construction materials and their flame retardancy. This review offers a comprehensive overview to researchers in the fields of fire engineering and material science, and it encourages them to explore and consider the use of AI in future research projects.

## 1. Introduction

Innovative materials nowadays have a significant impact on our daily lives and the industry. However, when considering if a new material can be used in the construction industry, its fire performance is one of the important factors that needs to be taken into account. As a result of several years of study, there are currently three fundamental methods for assessing the fire resistance of materials and their structural elements. The most classical and reliable method is the performance of an experiment in accordance with standards and regulations (i.e., the cone calorimetry test [1]). However, since many resources are needed, such as budget and time, to perform experiments, it is difficult to consider the influence of small changes and the significant variability of the parameters. The second method involves the use of empirical formulas based on the findings of the conducted fire tests. This approach is only suitable for materials of the same or similar composition as previously tested materials, and it cannot be applied to advanced materials. That is why numerical simulation (e.g., computational fluid dynamics (CFD) [2]) appears as the third approach that enables researches to carry out a larger number of analyses. Nevertheless, a major shortcoming is that the numerical model requires a very time-consuming process, extensive computational resources, and the dependence on a wide range of empirical parameters.

Nowadays, it is prevalent to apply artificial intelligence (AI) in different areas of life. Machine learning (ML), which is a subset of AI, has been widely used in image and speech recognition [3,4], web searches [5], fraud detection, [6], and email/spam filters [7]. Additionally, the utilization of ML in solving the scientific problems of areas including physics [8], chemistry [9], medicine [10], pharmacy [11], and biology [12,13] is thriving. Though the combination of ML and material sciences has just developed, several ML algorithms have demonstrated their ability to speed up the optimization and discovery of different functional materials for use in catalytic, photovoltaic, optical, and thermoelectric applications [14,15,16,17,18,19,20,21]. Unlike the CFD approach, ML uses a series of algorithms to construct statistical models to carry out predictions based on sample data instead of relying on existing knowledge [22,23]. ML-derived models show advantages over CFD-based models to simulate more complex problems without considering the true essence of the input–output relationship. In addition, the computational speed of ML models is much faster than that of CFD models. In a systematic review carried out by Naser [24], the positive potential of applying machine intelligence as an advanced technique to supplement experimental and simulation methods used within the field of fire engineering and sciences was provided. Artificial neural networks (ANNs), among the ML-based computational methods, have emerged as an effective mathematical method in solving many complex scientific and engineering problems [25]. In the fire engineering discipline, AI-based models employing an ANN and a genetic algorithm have been increasingly used to predict the temperature-related properties of a variety of materials (e.g., concrete, steel, timber, and composite). Therefore, this review focuses on the development of models derived from these two algorithms.

In this review paper, an overview of recent progress in applying artificial intelligence to predict the fire behavior of certain types of materials and structural components, including flame-retardant materials, is provided. This review paper includes five sections. In Section 1, an introduction of several existing methods and their limitations to assess materials’ fire performance is provided. In Section 2, a brief description of the operation of ML models is presented. In particular, the explanation of input variables and the evaluation metrics of ML models are included. In Section 3, the utilization of AI/ML to predict the temperature-related properties of construction materials (e.g., concrete, steel, timber, and composites) is elaborated. In Section 4, the existing applications of AI/ML to predict some characteristics of flame-retardant materials are discussed. The perspectives on the advantages, current challenges, and future developments of ML-based models applied to flame-retardant materials are proposed. Finally, conclusions are given in Section 5. It is expected that this review will bring benefits to material scientists and provide them with the motivation to explore and consider the ML application in their research projects.

## 2. Brief Description of Machine Learning

### 2.1. Operation of Machine Learning Models

ML is one of the most innovative methods in the material science area in recent years. This technique is different from conventional computing methods such as the fire dynamics simulator [26]. As for traditional methods, the necessary information such as boundary conditions, functions, and computational requirements are transmitted into the software, and computers help to run that software. Eventually, the expected result is shown in the software window. This method provides a high efficiency in cases that require a large amount of iteration in calculation. However, one challenge for the numerical model is that it must go through a time-consuming process of validation and the use of empirical parameters, which are difficult to be determined through analysis or testing. ML models, in contrast, provide a sufficient dataset, and a suitable algorithm can train the models to learn from known data or past experience in order to self-study to make predictions about unknown data without human intervention [27]. The ML approaches, which show the ability to model linear or nonlinear complex relationships without considering the essence of the relationships between the input and output, demonstrate advantages over conventional computational methods. The key steps when building a machine learning model include: collecting data to form a valid dataset, choosing relevant descriptors, splitting the dataset into the training and test sets, selecting an appropriate algorithm to build model on the training set, and evaluating the predictive power of the model by test set.

Constructing a material dataset is one of the main challenges when building an ML model. This dataset must be reliable and well-defined for the input and output parameters [28]. The material data can be collected from experimental results, the literature, or databases (i.e., structures and properties databases). Typically, descriptors are considered the input variables of ML models, such as the composition or properties of the material; therefore, the descriptor set must contain unique information. In the ML approach, the whole dataset can be used to build the ML model. However, to inspect the predictability of the proposed model, it is better to utilize data that the model has never been seen before. That is why a dataset is generally partitioned into two independent sets: the training set and the test set. The training set aims to build up the model, and the test set is employed to estimate the predictive power of the model. Algorithm selection plays a vital role in building machine learning models. Depending on the structure of the training data, ML can be classified into three groups: supervised learning, unsupervised learning, and semi-supervised learning [27]. Some ML algorithms that are commonly used in the development and production of advanced materials include multiple linear regression, support vector machines, decision tree, artificial neural network, ensemble learning, and clustering [27,29,30]. Finally, the predictive power of the model is assessed by the statistical method. In predicting material properties, the coefficient of determination (R^2^) and root mean square error (RMSE) are most widely used to measure the difference between the predicted values and the true values of an ML model.

### 2.2. Explanation of Descriptors

Descriptors play an essential role in constructing ML models because they are regarded as the input variables of models. Usually, data attributes generate the descriptors in one ML model. In addition, utilizing a mathematical transformation can produce new descriptors from other existing descriptors in many cases. If those produced descriptors are highly correlated to the output results of the ML approach, they are selected as the input parameters of the model. Descriptors can be classified into different categories, such as compositional descriptors, experimental descriptors, and topological descriptors. Compositional descriptors can be referred to as the composition of each component that constitutes the material. Experimental descriptors include all the parameters relating to experiments, e.g., temperature, pressure, the heat of reaction, the heat of combustion, reaction time, and the amount of the reactants and redundant parameters. Several properties regarding the texture of a material (e.g., surface area, volume, porosity, and pore size) can be assigned to the group of topological descriptors. Some set of descriptors used in a variety of material systems for various purposes in the fire engineering discipline are summarized in Table 1.

Constructing descriptors requires an in-depth understanding of the characteristics of the model and scientific problems [31]. Selecting appropriate descriptors is dependent on the specific issues that need to be solved, and enumerating all possible descriptors in one ML model is not an easy task. However, some general rules can be applied in the descriptor construction. First and foremost, descriptors shall define the materials; thus, each descriptor must include unique information, such as the material properties, composition, or structure of the material. It must be ensured that each entry is discrepant. For example, in the study carried out by Mukherjee and Nag Biswas [32], the stress of concrete at elevated temperatures was determined by a set of descriptors: strain (*ε*), temperature (*T*), elastic modulus (*E_T_*) at that temperature, compressive strength (*f*_c*T*_), and ultimate strain (*ε_ulT_*). Secondly, there should be a limitation on the number of descriptors. Too many descriptors makes a model more complex and thus hinders its ability to predict due to overfitting [33] and increases its computation time. As a good rule of thumb, the number of fitted variables in a model should be less than half the number of data points to prevent overfitting [34].

### 2.3. Evaluation of the Performance of AI/ML Models

Model assessment is necessarily carried out to determine the accuracy of a model in predicting material properties. In statistics, concerning regression problems, the R^2^ and root mean square error (RMSE) or mean square error (MSE) are commonly used to measure the difference between the predicted values and actual values of a target output. Let consider a dataset of size n. The actual properties of each data point are denoted by y_1_, y_2_, …, y_n_. The corresponding predicted values of those properties are ${\hat{y}}_{1}$, ${\hat{y}}_{2}$, …, ${\hat{y}}_{n}$, respectively.

The mean of these properties:

(1) $$\bar{y}=\frac{1}{n}\overset{n}{\underset{i=1}{\sum }}y_{i}$$

Total sum of squares:

(2) $${\mathrm{SS}}_{\mathrm{tot}}=\sum_{i=1}^{n}{\left(y_{i}-\bar{y}\right)}^{2}$$

Residual sum of squares:

(3) $${\mathrm{SS}}_{\mathrm{res}}=\sum_{i=1}^{n}{\left(y_{i}-{\hat{y}}_{i}\right)}^{2}$$

The R^2^ can be determined as follows:

(4) $$R^{2}=1-\frac{{\mathrm{SS}}_{\mathrm{res}}}{{\mathrm{SS}}_{\mathrm{tot}}}$$

RMSE is calculated as below:

(5) $$\mathrm{RMSE}=\sqrt{\frac{\sum_{i=1}^{n}{\left(y_{i}-{\hat{y}}_{i}\right)}^{2}}{n}}$$

The use of R^2^ in conjunction with RMSE or MSE to evaluate the prediction capability of a model is strongly recommended [35,36]. It is suggested that a well-defined regression model should have an R^2^ value close to 1.0 and an RMSE value close to 0 [36].

If an ML model can make accurate predictions on unseen data, it is supposed to have the ability to generalize from the training set to the test set. In other words, it possesses generalization capability. The model’s performance might suffer from underfitting or overfitting. If a too-simple model with inadequate descriptors is built, it will cause underfitting, which means the model will perform poorly on both training and test sets. More descriptors should be placed in the model to tackle this problem and improve the prediction ability. Some new descriptors can be generated by a mathematical transformation from the current set of descriptors. In contrast, overfitting occurs when an ML model is too complicated and has too many descriptors. In this case, the model works very well on the training set but makes a worse prediction or poor generalization on the test set. The typical approach to address this issue is using a training set that accounts for a certain proportion of the dataset, ranging from two-third to 80%. The remainder of the dataset is allocated to the test set. The more similar the training set and test metrics are, the more reliable the model is. This approach seems realistic and straightforward, but it requires that the original dataset must be sufficiently large in order to produce good results. This strategy is the so-called train-and-test method. If the size of the dataset is large enough, it can even be divided into three sets: training, validation, and test sets. The function of the validation set is to select the best parameters of the ML model. After that, one can rebuild a model with the parameter setting that was found by training on both the training and validation data. Moreover, some other techniques can help prevent overfitting, including minimizing the number of descriptors by eliminating the weakly correlated ones or increasing the dataset size.

In addition to the train-and-test method, one technique can be applied to the relatively small dataset for assessing the accuracy of the model, which is cross-validation. The detail and the variation of this technique can be found in a paper by Arlot and Celisse [37]. When performing cross-validation, the original dataset is firstly partitioned into k parts of approximately equal size, called folds. The training and testing process is then performed k times. Each time, the model is constructed using the data in (k-1) folds, and the model accuracy is evaluated on the remaining fold. This process is often called k-fold cross-validation.

## 3. Application of AI/ML in Fire Engineering

In the fire engineering discipline, great attempts have been made to evaluate the behavior of construction materials, such as concrete [38,39,40,41,42,43,44,45], steel [46,47,48,49,50,51,52,53], timber [54,55,56,57,58], and composites [59,60,61,62,63,64,65,66,67], exposed to fire conditions by conducting experimental programs. However, since many resources, such as budget and time, to perform experiments are required, AI-based models have emerged as powerful tools used to predict the temperature-related properties of a variety of materials. The behavior of some types of structural systems, e.g., columns, beams, frame, trusses, or joints exposed to high temperature, has also been investigated by the application AI-based models, typically ANNs.

### 3.1. Concrete Elements/Structures

**Figure 1:**
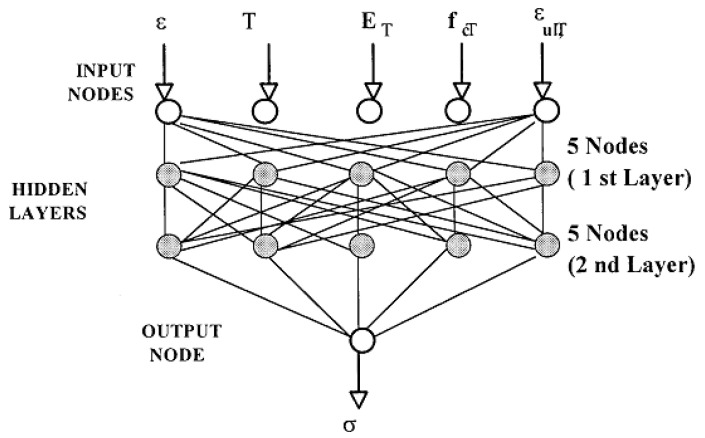
The structure of a feedforward network. Adapted with permission from ref. [32]. Copyright 1997 Open Access & Nature Research.

Since concrete is a composite material composed of cement and aggregates, the mechanical behavior of concrete under fire conditions is nonlinear, extremely complicated, and highly temperature-dependent. It is tough to incorporate all contributing factors in a mathematical model to obtain concrete behavior under elevated temperatures. To overcome this difficulty, ANNs have been employed to predict concrete element fire performance. A neural network consists of three layers, as shown in Figure 1. There is an input layer includes the nodes corresponding to the number of descriptors, followed by one or some hidden layers, and, finally, an output layer containing the target properties to be predicted. The weights between each layer of the network are modified during the computational process to produce the best match to the given data. It can be noticed that different input descriptors come with different scales of their values. Thus, the data should be normalized to a range between 0 and 1 for all descriptors before training the model. In the end, the output production of the model is also within this 0–1 range. With the advantage of modeling complex nonlinear, multi-functional engineering problems over other machine learning algorithms, ANNs have attracted considerable attention from the scientific community in evaluating the fire performance of concrete material and concrete structural elements.

One of the earliest studies to apply AI in predicting the properties of concrete material under high temperature was conducted by Mukherjee and Nag Biswas [32]. A feedforward and backpropagation algorithm was employed and integrated into the ANN model to obtain the stress–strain relationship of concrete under three different cases: varying load under isothermal conditions (case 1), varying temperature under constant load (case 2), and varying temperature under total restraint (case 3). For case 1, strain (*ε*), temperature (*T*), elastic modulus (*E_T_*) at that temperature, compressive strength (*f*_c*T*_), and ultimate strain (*ε_ulT_*) were considered as input descriptors, with the target output as the stress being depicted in Figure 1. An RMSE value of 0.000484 demonstrated a strong agreement between the measured data and the predicted result. In case 2, another set of input descriptors was applied, including temperature (T), load level (h), modulus of elasticity (*E_T_*), compressive strength (*f*_c*T*_), ultimate strain (*ε_ulT_*), and the coefficient of the thermal expansion (Ω_T_); the output of the ANN model was strain (*ε)*. Finally, in case 3, the six input parameters of the model were temperature (T), modulus of elasticity (*E_T_*), compressive strength (*f*_c*T*_), ultimate strain (*ε_ulT_*), thermal expansion coefficient (Ω_T_), and heating rate (λ), while the target output was set to be the restrained stress (σ). In both cases 2 and 3, the neural network proved its capability to learn from experimental observation and make very good predictions.

Chan et al. [68] developed an ANN-based model to predict the degradation of the compressive strength of concrete exposed to fire conditions (75–1200 °C). With target as the loss of concrete strength, the input nodes of the model contained variables relating to experimental parameters and environmental factors. The prediction error within 15% between experimental and analytical results indicated the potential in adopting this advanced approach in the concrete analysis. The use of ANNs in predicting compressive strength degradation of self-compacting concrete (SCC) under elevated temperature was also investigated in another study by Uysal et al. [69]. While setting the loss in compressive strength as the targeted output, a set of compositional constituents and the heating degree was assigned as the input variable of the neural network. The ANN model was built on the dataset with the size of 85 data samples. The numbers of data points allocated for the training set and test set were 43 and 42, respectively. The R^2^ coefficient of 0.9757 demonstrated the excellent prediction capability of this advanced programming approach in fire engineering.

**Figure 2:**
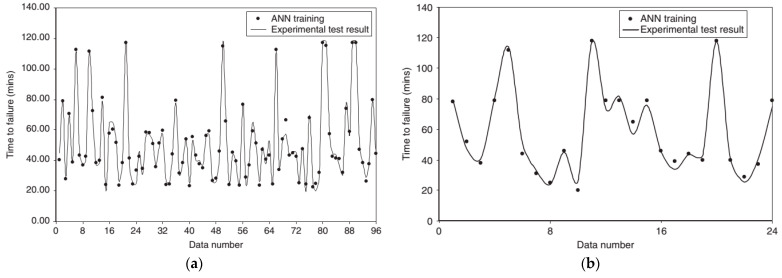
Comparison of experimental data and an artificial neural network (ANN) model to predict the failure time of concrete columns on a (**a**) training set and (**b**) test set. Adapted with permission from ref. [70]. Copyright 2014 Journal of Structural Fire Engineering.

An ANN also possesses power in predicting the performance of concrete columns exposed to high temperatures. McKinney and Ali [70] applied the supervised ANN method to classify the temperature-induced spalling phenomenon and predict the failure time of concrete columns. In this research, two neural network models with architectures of 6–10–3 and 5–10–1 (input nodes–hidden nodes–output nodes) were employed for spalling classification and failure time prediction, respectively. Among 30 test results selected for the model construction, 80% of the dataset were allocated to the ANN training process, and the remaining 20% were retained for the test set. As a result, 0.987 and 0.983 were the R^2^ scores obtained for estimating failure time in the training set and test set, respectively, while the case of the concrete spalling classification produced the outcomes with an error of 7%. A comparison between the experimental observations and ANN modeling results is shown in Figure 2. All this demonstrated the ANN’s capability to assess the fire performance of high-strength concrete columns. The fire resistance of concrete columns under fire conditions also drew interest from the fire engineering scientific community. Some research groups have successfully constructed and applied ANNs to predict this characteristic of concrete columns [71,72]. In these studies, the ANN models set the fire resistance of columns as the output, while the input variables were dimensional descriptors and loading conditions. All aforementioned models proved the predictive powers with almost-zero RMSE value or an error within an acceptable range.

Concrete spalling is not a new phenomenon and has been interpreted by carrying out experimental studies. It is generally the explosion at the surface layers of the concrete element exposed to an elevated temperature that could be triggered by fire. However, by leveraging the power of artificial intelligence and machine learning, Naser constructed many AI-derived models to predict the fire-induced spalling and fire resistance of concrete elements [73,74,75,76,77,78]. These models were built on the basis of various machine learning algorithms (e.g., logistic regression, decision tree, random forest, gradient boosted trees, and support vector machine) or the combination of neural networks with genetic algorithms. For spalling classification, the target of these AI-based models was the binary output consisting of “non-spalling” and “spalling.” The failure temperature or failure time could be set as the expected outputs for the fire resistance of concrete elements, while the input parameters of these models were dimensional descriptors, loading conditions, or mechanical properties of concrete. All models developed by Naser proved the predictive powers, resulting in an error within the acceptable range and an R^2^ coefficient close to 1.0. These aforementioned studies demonstrated the merit of utilizing numerous AI approaches to develop reliable models capable of predicting fire-induced spalling phenomenon and fire resistance of concrete structural elements with high accuracy.

**Figure 3:**
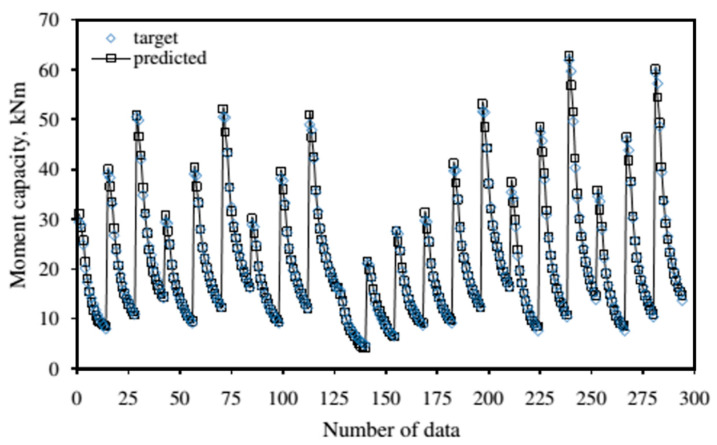
Comparison of the calculated flexural capability with the predicted flexural capability. Adapted with permission from ref. [79]. Copyright 2009 Advances in Engineering Software.

In another notable research, Erdem [79] constructed an ANN model based on 294 experimental data to estimate the maximum moment capacity of reinforced concrete slabs under fire conditions. 206, 44, and 44 data points were allocated for the training, validation, and test sets, respectively. The proposed model contained seven input parameters relating to the dimensional aspect of concrete slabs and some mechanical properties of concrete and its reinforcement. As a result, the correlation coefficients in the training, validation, and test sets were 0.99775, 0.99795, and 0.99750, respectively. It was stated that the proposed model was fit for the determination of flexural capability of concrete slab exposed to fire by providing a good generalization with a high degree of accuracy. A comparison of the calculated results with the predicted values of moment capacity is presented in Figure 3.

The addition of polymeric fibers to a concrete structural element not only strengthens its mechanical properties but also allows them to be more fire-resistant by reducing the risk of spalling. To continue to apply the power of AI in the development of numerical models, Altun et al. [80] launched a study to investigate the fire performance of a prismatic concrete beam with the effect of adding polypropylene fibers. In this study, three types of AI-models, namely multilayer perceptron (MLP), an adaptive neuro-fuzzy-inference-system (ANFIS), and a fuzzy-genetic model, were used to predict toughness; 216 out of 432 samples from the experiment were randomly selected to build up the AI-based models, in which 60%, 20%, and 20% of 216 points data were, respectively, allocated for the training, validation, and test sets. Different parameters relating to fiber types, fiber ratios, curing periods, and temperature effects were taken into account as the input descriptors of the ANN model. As a result, the fuzzy-genetic model was the most successful by producing a mean absolute relative error value of 7.945% compared to 10.253% and 11.226% for the MLP and ANFIS models, respectively. It was also revealed that the input–output relationship that is inherently difficult to model could be successfully obtained by utilizing AI-based models. In another study, Naser et al. [81] developed an ANN model to evaluate the fire resistance of T-shaped reinforced concrete beams under different fire scenarios. In the experimental program, these beams were strengthened by carbon fiber-reinforced polymers (CFRPs) and insulated by various protective materials [82]. Since the validated finite element (FE) model developed by Hawileh et al. [83] and the experimental data were matched, the FE model was used as a benchmark to generate additional data points to train the ANN. The dataset with 120 data points was split into the training set (90 data points) and test set (30 data points). The output of the developed ANN model was the temperature between CFRP and concrete interface, while the main input parameters consisted of insulation thicknesses, types of material, and fire curves. The developed ANN model was able to achieve an excellent matching score (R^2^ of 0.9805) with the observed data and validated FE model.

### 3.2. Steel Elements/Structures

In addition to its use for concrete structures, an ANN was also successfully applied when making predictions and assessing the fire-induced behaviors of steel structures or steel structural components. Naser had successfully developed an ANN tool that is useful for deriving the temperature-dependent properties of structural steels [84]. The model construction was based on the data collected from the literature and fire codes or standards (American Society of Civil Engineers, Eurocode 3, British Standard, etc.). Seventy percent of the dataset were allocated for training the model, and the remaining 30% was used to test the predictability of the neural network. The thermal properties and reduction factor of some mechanical properties of the structural steel were derived as the temperature-dependent functions owing to the utilization of the ANN combined with genetic algorithms. The developed model obtained R^2^ values of 0.998, 0.901, 0.995, and 0.983 in the cases of the thermal conductivity, specific heat, yield strength, and elastic modulus properties, respectively. The AI approach was found to be adequate for the derivation of temperature-dependent thermal and mechanical properties of steel materials.

The power of AI, specifically the ANN, continued to be demonstrated by research carried out by Hozjan et al. [85]. An ANN model with an architecture of 2–50–50–1 (two neurons in the input layer, 50 neurons in each hidden layer, and one neuron in the output layer) was employed to determine the stress of structural steel exposed to fires based on the consideration of strain and temperature as the input variables. In their study, 527 data points were divided into a training set with a size of 435 and a test set with 92 data points. In this case, the developed model gave a very high R^2^ value of 0.9993. Though the predicted results were in line with the experimental observations, there were some obstructions in constructing the ANN model. For instance, in the range of strains greater than 2%, the ANN model was insufficient; therefore, it was required to add a constant hardening parameter in this range. Another problem was that the predicted stress–strain relationship under the yield limit deviated from a linear form because of ANN regression. A linear regression based on experimental measurement should be applied for this range, assuming an ideal linear behavior of steel. However, the usefulness of using an ANN in predicting the property of structural steel exposed to high temperatures was proven.

The mechanical behavior of the tubular truss was not easy to capture due to the dependence on a large number of affected parameters. While the FE method was found to be ineffective, AI techniques emerged as a powerful tool to address these problems. In a study conducted by Jixiang Xu et al. [86], an ANN with the structure of 4–10–1 for input–hidden–output nodes was employed to predict the limiting temperature of steel tubular truss under fire conditions. The neural network input descriptors included geometrical parameters of the web and chord members that made up the truss. The developed ANN model used 105 input–output pairs for the training process and 15 sets of data for testing its predictability. Providing an R^2^ score of 0.99946 for the training set and 0.9975 for the test set pointed out that the performance of the neural network was excellent. However, getting satisfactory ANN results required a lot of time to process the dataset construction. Additionally, the model predictability depended primarily on the accuracy level in the training set.

The strength of AI continued to be reflected in a study carried out by Zhao [87]. A combination of AI techniques, including a backpropagation neural network, a radial basis function neural network, and a genetic algorithm (GA), was employed to estimate the failure temperature of steel columns under high temperatures. Some geometrical parameters of steel columns were considered as the input variables of the AI-based model. Two-thirds of the total 102 sets of experimental data were chosen for the training set, and one-third with 34 data points was selected for the test set. It was found that the proposed AI model was better than the modified Rankine method in terms of accuracy level by comparing mean-square-error values. Some advantages could be drawn by the utilization of this developed AI model. Firstly, it saved lots of computing time to predict the target output given the input descriptors owing to neural networks. Secondly, the GA helped to manage the model’s complexity. Finally, additional experimental data could be easily integrated into the existing model if available, improving the model accuracy. Nevertheless, a disadvantage of this model was that incorporating the GA made the model quite time-consuming, thus requiring significant improvements.

### 3.3. Timber Elements/Structures

There have been some remarkable studies that have demonstrated the power of AI in predicting some temperature-dependent properties of timber material and structural components. ANN models were developed by Cachim to estimate the temperature of timber exposed to fires [88]. The three input descriptors of the model were timber density, fire exposure time, and the distance from the measuring point to the exposed surface. With a total of 41 data points, 30% of the dataset were selected for training the network, and the rest of the dataset was used for evaluating the prediction capability of the model. A parametric study was carried out by changing the number of hidden layers (one, two, or three) and the number of nodes in each hidden layer (five, seven, or nine). The network with two hidden layers, which included five and seven neurons in the first and second layers, was found to be the best-constructed model. It provided an R^2^ score of 0.9998 and an RMSE value of 3.3. The model outputs were also compared with the results produced by the numerical model SAFIR, which is a thermal/structural program for structure analysis under fire conditions. The developed model was reported to be reliable in calculating the temperature in timber elements.

In another study, Naser [89] succeeded in integrating an ANN with symbolic regressions and genetic algorithms to generate a robust AI-based model to derive temperature-dependent expressions at the material and elemental levels of timber under fire conditions. Databases with over 12,000 data points for model construction were gathered from the conducted fire tests, fire design codes, and standards from the literature review. At the material level, the mechanical and thermal properties of wood (i.e., Young’s modulus, compressive strength, tensile strength, shear strength, thermal conductivity, specific heat, and charring depth) were expressed as a function of temperature. Additionally, at the elemental level, some thermo-structural responses of timber components (e.g., floors, beams, columns, and connections) were also determined based on the consideration of related compositional, dimensional descriptors, and loading conditions. Further details on the input–output parameters used in this study can be found in Table 1. For all cases, the almost-one values of R^2^ and nearly-zero values of the mean absolute error proved the strength of using the combination of an ANN, symbolic regression, and genetic algorithms for deriving the expressions of the temperature-dependent properties of wood, as well as for tracing the thermal-structural responses of timber elements.

Lautenberger et al. [90] employed a genetic algorithm to generate inputs for computational fluid dynamic models to evaluate the fire-induced properties of redwood and red oak under fixed heat flux in a cone calorimeter. In this study, eight properties of wood in non-charring and charring phases (i.e., thermal conductivity, specific heat, pre-exponential factor, activation energy, the heat of pyrolysis, char thermal conductivity, char specific heat, and char density) were required as the input parameters for pyrolysis modeling developed by fire dynamic simulation (FDS) software. A set of these above-mentioned properties was firstly obtained by a GA and then transmitted to the FDS pyrolysis model. At the end, the mass-loss rate and surface temperature of redwood and red oak could be expected as the final results. This process was complicated due to the involvement of both the GA and pyrolysis modeling because they were normally time-consuming. One noticeable point was that the properties found by the GA were understood as the average values over the different test conditions and assumed to be temperature-independent for the modeling simplicity. These properties might not be applied in another pyrolysis model based on other assumptions to simplify the model. However, a good agreement between the experimental observations and model prediction proved the capability of this methodology to obtain the mass loss rate and surface temperature of timber material.

### 3.4. Composite Materials

A few studies have utilized a GA to predict the temperature-dependent properties of composite materials. Rein et al. [91] succeeded in applying this AI technique to estimate the kinetic parameters required for theoretical modeling of the smoldering combustion of flexible polyurethane (FPU) foam. Firstly, based on the outcomes from previous thermogravimetric analyses, a global mechanism of FPU five-reaction kinetics was proposed. Secondly, the development of a numerical lumped model of mass loss for FPU was carried out to simulate the thermogravimetric experiment. Finally, the GA technique was employed to find a set of kinetic parameters, including activation energy (E), pre-exponential factor (A), power-law parameter for reaction (n), and mass yield/consumption of species per mass of reactant in the reaction (ν) that provided the best agreement between the numerical results and experimental observations. The result obtained by the GA was found to help model the numerical simulation of smoldering combustion.

The genetic algorithm is a robust technique inspired by Darwin’s theory of natural evolution [92]. The genetic algorithm is initialized by the creation of a population consisting of a number of randomly candidate solutions. Each candidate solution is referred to as an individual among the population. Each individual is defined by a set of parameters known as genes. For instance, in the above-mentioned study conducted by Rein et al. [94], each unknown kinetic parameter (i.e., E, A, n, and ν) was a so-called gene for an individual in the GA population. The continual evolvement of populations resulted in subsequent generations. The first generation was the original population, the second generation was the offspring of the first generation, and so on. The processes of mutation, crossover, and reproduction were included in this approach, and further information can be found in [95]. These processes were repeatedly continued until the convergence of candidate solutions.

The GA was also used as a potential tool to generate the required input parameters for CFD-based models that simulate the combustion process of composites under the cone calorimeter test [90,93]. In a study carried out by Lautenberger et al. [90], a set of eight parameters, consisting of thermal conductivity, specific heat, pre-exponential factor, activation energy, heat of pyrolysis, char thermal conductivity, char specific heat, and char density, was generated by the GA. These parameters were then used in CFD models to perform numerical analyses. Yuen et al. [96] applied the power of the GA to find out a pool of input variables for the CFD-based fire growth model, including composition (c_i_), pre-exponential factor (A_i_), activation energy (E_i_), and exponent (n_i_). The final results obtained by the numerical model were surface temperature and mass loss rate of polypropylene [90], or heat release rate, total heat release, smoke production rate, and total smoke production of flame-retardant composites [93] under a bench-scale fire test. Though the combined GA/pyrolysis model was found to provide a good agreement between predicted and measured data, it did not help save computing time and computational resources, as two analyses were required: one for the GA and another for the pyrolysis model.

### 3.5. Other Types of Elements/Structures

Another piece of evidence to demonstrate the strength of the ANN was found by applying this advanced approach to assess the behavior of semi-rigid composite connections under fire conditions. In a study conducted by Al-Jabri et al. [94], the rotation of beam-to-column-joints was predicted based on the designation of the applied moment and the temperature of the joints as the input variables of the ANN model. The dataset was constructed based on the results of 20 fire tests, in which 10–15% of the data were assigned to the test set, and the rest of the data was used for the training set. Consequently, by generating R^2^ values in a range from 0.926 to 0.983 for the training set and between 0.896 and 0.993 for the test set, it was concluded that the model results were in line with the experimental observations. The rotational response of semi-rigid joints in the fire event was also investigated by Al-Jabri et al. in another study [95]. However, the dimensional factors, mechanical properties of the joints, temperature, and loading conditions were designated as the input descriptors for the ANN model. The dataset of 280 cases obtained from nine fire experiments was divided into the training set with 236 cases, and the test set was composed of 44 cases. The R^2^ scores for the training set and the test set were 0.998 and 0.970, respectively. A trial to construct other ANN models was recommended by considering parameters (e.g., axial restraints and temperature gradient) that significantly impacted the joint’s rotation. Both aforementioned studies proved the model’s capability of predicting the performance of composite joints under fire scenarios.

Neural networks were developed to become superior alternatives that save computational resources and computing time compared to the CFD approach for predicting important parameters in a single compartment fire [96,97]. One downside of an ANN was that the noise contained in the dataset cannot be differentiated from the genuine features in the network training process. Therefore, instead of utilizing an ANN, these studies proposed an AI technique based on general regression neural network (GRNN) and fuzzy adaptive resonance theory (FA), with the so-called general regression neural network with fuzzy adaptive resonance theory model (GRNNFA) used to predict the location of thermal interface [99] and the velocity and temperature profiles at the center of the doorway [97] in a single compartment fire. While FA was applied to generate prototypes for network training based on the training data distribution in the input domain, the GRNN was employed for prediction. The input variables of the network included six important parameters, such as the dimensional factors relating to the opening, fire strength, and temperature. The proposed GRNNFA model was built on the basis of a dataset of 55 experimental fire tests. In the case of predicting thermal interface location, only 3 out of 55 samples were incorrectly predicted, and the accuracy level was 94.5%. In the prediction of the velocity and temperature profiles, the prediction errors of the GRNNFA model fell within the acceptable range and were smaller than those of the CFD model. The accuracy of the model was also found to be linked to the number of data points given for network training. These results proved the potential of the utilization of the GRNNFA to predict compartment fire parameters.

Further details on the applied method and descriptor set of AI-based models, used to predict the properties of different types of material exposed to fire and evaluate the thermally-induced responses of a variety of structural components, can be found in Table 1.

**Table 1:** Typical descriptors used in artificial intelligence (AI)/ML (machine learning) models to predict the fire-induced properties of some types of structural elements.

Type of Structure	Method	Target Output	Descriptors/Input Parameters	Reference
Concrete material	Artificial neural networks	The stress (σ)	Strain (*ε*), temperature (*T*), elastic modulus (*E_T_*) at that temperature, compressive strength (*f*_c*T*_), and ultimate strain (*ε_ulT_*)	[32]
Concrete material	Artificial neural networks	The strain (ε)	Temperature (T), load level (h), modulus of elasticity (*E_T_*), compressive strength (*f*_c*T*_), ultimate strain (*ε_ulT_*), and the coefficient of the thermal expansion (Ω_T_)	[32]
Concrete material	Artificial neural networks	The restrained stress (σ)	Temperature (T), modulus of elasticity (*E_T_*), compressive strength (*f*_c*T*_), ultimate strain (*ε_ulT_*), thermal expansion coefficient (Ω_T_), and heating rate (λ)	[32]
Concrete material	Artificial neural networks	The loss of strength	Experimental parameters and environmental factors	[68]
Self-compacting concrete	Artificial neural networks	The compressive strength	The amount of cement, fly ash, zeolite, limestone powders, basaltic, marble powders, natural aggregate, group I aggregate, group II aggregate, polypropylene fibers, heating degree	[69]
High-strength concrete columns	Artificial neural networks	The spalling type	Furnace temperature, restraint, loading level, force, spalling degree, failure time, and spalling type	[70]
High-strength concrete columns	Artificial neural networks	The failure time	Furnace temperature, restraint, loading level, force, spalling degree, and failure time.	[70]
Reinforced-concrete columns	Artificial neural networks	The fire resistance of the column expressed in minutes (t)	Dimensions of the cross-section of the column (b and d), the concrete cover thickness (a), percentage of reinforcement (μ), load coefficient for axial force (η), and load coefficient for the bending moment (β).	[72]
Concrete-filled tubular steel columns	Backpropagation neural network	The fire resistance	Structural factors (external dimension, steel thickness, column height), material factors (water–cement ratio, type of aggregate, concrete 28 days cylinder strength, steel yield strength), loading conditions (test load)	[71]
Concrete slabs	Artificial neural networks	The ultimate moment capacity (M_u_)	The distance from the extreme fiber in tension to the centroid of the steel on the tension side of the slab (d’), the effective depth (d), the ratio of previous parameters (d’/d), the area of reinforcement on the tension face of the slab (A_s_), the fire exposure time (t), the compressive strength of the concrete (f_cd_), and the yield strength of the reinforcement (f_yd_)	[79]
Prismatic concrete beams	Artificial neural network, fuzzy logic, and fuzzy genetic models	The toughness (T_g_) value of the prismatic beams	The fiber type used to prepare the specimen mixtures (F_t_), curing period (C_p_), temperature (T), volumetric fiber ratios in the mixture (F_R_), the compressive strength of the cylindrical specimens (f_c_)	[80]
Reinforced-concrete T-beams strengthened with carbon fiber-reinforced polymer (CFRP) plates	Artificial neural networks	The temperature at the interface between the CFRP/concrete	Insulation thicknesses, materials types, and fire curves	[81]
Structural steel	Artificial neural networks and genetic algorithms	Temperature-dependent material properties: thermal conductivity, specific heat, reduction factor for yield strength, and reduction factor for modulus of elasticity	Temperature	[84]
Steel frames	Artificial neural networks	The stress (σ)	Strain (*ε*) and temperature (T)	[85]
Steel tubular truss	Artificial neural networks	The limiting temperature	Diameter ratio (*β*), the wall thickness ratio (*τ*), the diameter–thickness ratio (γ), and the load ratio	[86]
Steel columns	Hybrid neural network and genetic algorithm	The failure temperature (T)	The length of the steel columns (L), the radius of Gyration of the cross-section (r), the sectional area (A), the yield strength of the material at room temperature (f_y_), the applied load (P), and the eccentricity of the load at failure (e)	[87]
Timber member	Artificial neural networks	The temperature in timber	The density of timber, the time of fire exposure, and the distance from the exposed surface	[88]
Timber material	Artificial neural network together with symbolic regressions and genetic algorithms	Mechanical properties (Reduction factors of density, Young’s modulus, compressive strength, tensile strength, and shear strength), thermal properties (thermal conductivity and specific heat), charring depth	Temperature	[89]
Timber floors	Artificial neural network together with symbolic regressions and genetic algorithms	The temperature in the plywood subfloor	Fire exposure duration (t), number of layers in ceiling finish (C), sub-floor thickness (k_th_), and type of cavity insulation (I)	[89]
Timber beams	Artificial neural network together with symbolic regressions and genetic algorithms	Mid-span deflection	Fire exposure time (t), load level (P), height (H), and charring rate (β)	[89]
Timber columns	Artificial neural network together with symbolic regressions and genetic algorithms	Fire resistance expressed in minutes	Column depth (D), column breadth (B), compressive strength (f_c_), specific gravity (S_G_), and level of applied loading (P)	[89]
Finger–joint timber connections	Artificial neural network together with symbolic regressions and genetic algorithms	Fire resistance expressed in minutes	The adhesive type (A), width (W), charring rate (β), and applied loading (P)	[89]
Nailed timber connection	Artificial neural network together with symbolic regressions and genetic algorithms	The slip of the connection (d)	Fire exposure time (t) and load level (P)	[89]
Redwood and Red Oak	Genetic algorithms and pyrolysis model	Surface temperature and mass loss rate	Thermal conductivity (k_v_), specific heat (c_v_), pre-exponential factor (Z), activation energy (E_A_), and heat of pyrolysis (ΔH_p_), char thermal conductivity (k_c_), char specific heat (c_c_), and char density (𝜌_c_)	[90]
Flexible polyurethane foam (Composite material)	Genetic algorithms	Kinetic and stoichiometric parameters	The reaction mechanism of thermal and oxidative degradation with thermogravimetric data	[91]
Polypropylene (Composite material)	Genetic algorithms and pyrolysis model	Surface temperature and mass loss rate	Thermal conductivity (k_v_), specific heat (c_v_), pre-exponential factor (Z), activation energy (E_A_), and heat of pyrolysis (ΔH_p_), char thermal conductivity (k_c_), char specific heat (c_c_), and char density (𝜌_c_)	[90]
Chitosan/graphene oxide layer-by-layer fire retardant coating on flexible polyurethane foam (composite material)	Genetic algorithms and computational fluid dynamics model	Thermal degradation rate, heat release rate, total heat release, smoke production rate, total smoke production, CO production rate, total CO production	Composition (c_i_), pre-exponential factor (A_i_), activation energy (E_i_), and exponent (n_i_)	[93]
Semi-rigid beam-to-column joints	Artificial neural network	The rotational capacity of the joint	The applied moment and joint’s temperatures	[94]
Semi-rigid composite joints	Artificial neural network: Backpropagation paradigm	The rotational capacity of the joints	The joint geometrical properties (beam depth, beam width, beam flange thickness, beam web thickness, column depth, column width, column flange thickness, column web thickness, number of bolts, bolt diameter, end-plate thickness, end-plate depth, end-plate width), the joint mechanical properties (beam yield strength, column yield strength), the joint’s temperature, and the applied moment	[95]
A single compartment fire	General regression neural network and fuzzy adaptive resonance theory	The location of the thermal interface, the height of the thermal interface, and different widths of the opening	The width and height of the sill of the opening, parallel and perpendicular distances from the center of the fire bed to the vertical centerline of the opening, fire strength, and ambient temperature	[96]
A single compartment fire	General regression neural network and fuzzy adaptive resonance theory	The velocity and temperature profiles at the center of the doorway	Width of opening, the height of the sill of the opening, fire strength, distance from the vertical centerline of the opening to the center of the fire bed, distance from the vertical centerline of the opening to the center of the fire load, and the ambient temperature	[97]

## 4. Application of AI/ML for Flame-Retardant Materials

### Existing Studies

**Figure 4:**
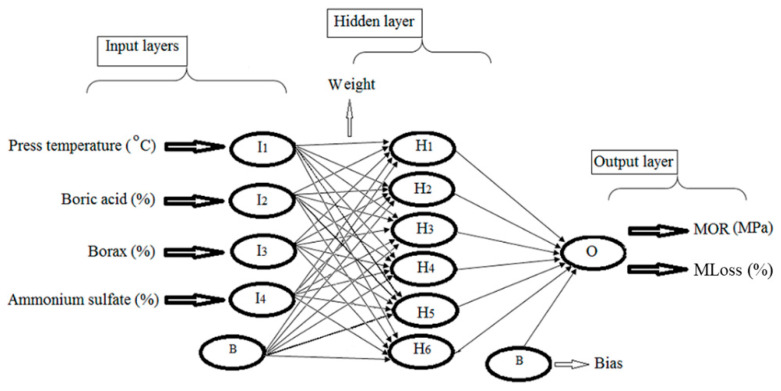
The structure of the ANN model to compute the fiberboard properties under fire conditions. Adapted with permission from ref. [98]. Copyright 2020 Open Access & Springer.

In a study conducted by Nazerian et al. [98], an ANN demonstrated its prediction capability in the estimation of the modulus of rupture (MOR) and mass loss (MLoss) of flame-retardant fiberboard. The researchers applied the response surface methodology and central composite rotatable design to prepare the experimental design. The measurement of the MOR and MLoss of the test specimens was followed by EN 310 (1993) Wood-based panels - Determination of modulus of elasticity in bending and of bending strength, and ISO 11925-3 (1997) Reaction to fire tests - Ignitability of building products subjected to direct impingement of flame - Part 3: Multi-source test, respectively. Three types of flame retardants consisting of boric acid, borax, and ammonium sulfate were involved in the study to investigate their effect on the MOR and MLoss of fiberboard during the fire test. The input parameters of the neural network model were the three above-mentioned mentioned flame-retardants at five different levels (0, 1.5, 3, 4.5, and 6%) and press temperatures at five levels (135, 150, 165, 180, and 195 °C). The target outputs included the fiberboard’s MOR and MLoss under fire conditions. Expert Design Software version 6 and the second-order plan statistical design were employed to estimate the effect of the model’s input variables on the output. Figure 4 describes the skeleton of the ML model applied in this study.

**Figure 5:**
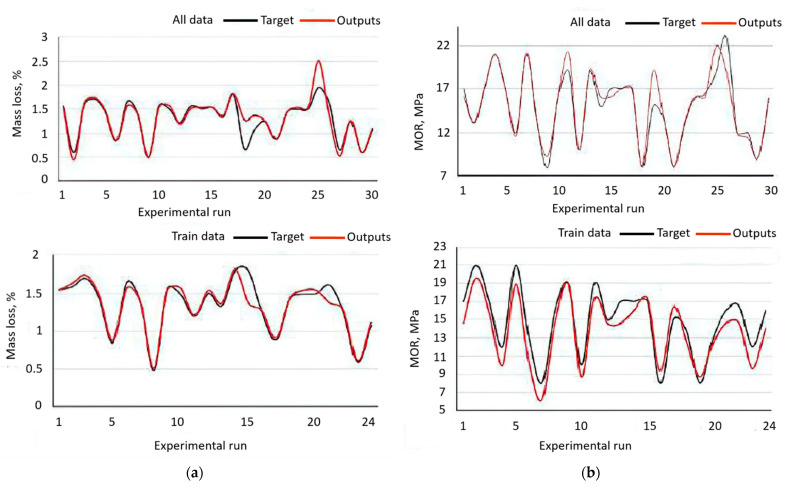
A comparison between the actual values (target) and predicted values (outputs) of the (**a**) Mass loss and (**b**) Modulus of Rupture in the cases of all data and train data. Adapted with permission from ref. [98]. Copyright 2020 Open Access & Springer.

Nazerian et al. selected a 90 sample data pool to perform an ANN model. The processes of training, validating, and testing the predictability of neural networks employed 70%, 15%, and 15% of the total data, respectively. Table 2 shows the statistical results of the ANN models in this study in terms of R^2^ and RMSE. The prediction error was shown to be within a reasonable range and R^2^ was close to 1, thus indicating a strong agreement between the ANN model and the experimental outcomes. A comparison between the actual and predicted values of the ANN model in terms of the MLoss and MOR is illustrated in Figure 5. The predicted result from the neural network was similar to the actual values for both the MLoss and MOR. Therefore, it was concluded that the ANN model was capable of predicting the MLoss and MOR with a high accuracy.

The effect of flame retardants on model construction and model outputs was another crucial aspect. The fiberboards in this study were made up of resin, fiber, and flame-retardants. Since the resin and fiber contents were the same for all specimens, flame-retarding agents at five different contents (0, 1.5, 3, 4.5, and 6%) had to be considered as the input parameters of the neural network in addition to the press temperature. In other words, the ANN model would not have been adequately constructed without the proper determination of these parameters. As a result of Expert Design Software version 6 and the second-order plan statistical design, the press temperature (x_1_), boric acid (x_2_), borax (x_3_), and ammonium sulfate (x_4_) were found to have an essential impact on MLoss, while x_4_ showed a minimal effect on the MOR. Furthermore, while the MLoss and MOR were substantially influenced by the squared values of x_2_ and x_3_, they did not show the reliance on the squared value of x_1_. The squared effect of x_4_ indicated the significant impact on MLoss, but it was not influential on the MOR. Moreover, MOR values were considerably dependent on the mutual impacts of x_1X2_, x_2X3_, and x_3X4_, while MLoss also relied on the mutual effects of x_1X3_ and x_2X4_. It was also observed that the fire rate decreased as the content of fire retardant chemicals increased, and, as a consequence, the mass loss decreased. To sum up, adopting the proposed ANN model could save time and resources to perform experiments, while the desired output of the MLoss and MOR could be achievable.

**Figure 6:**
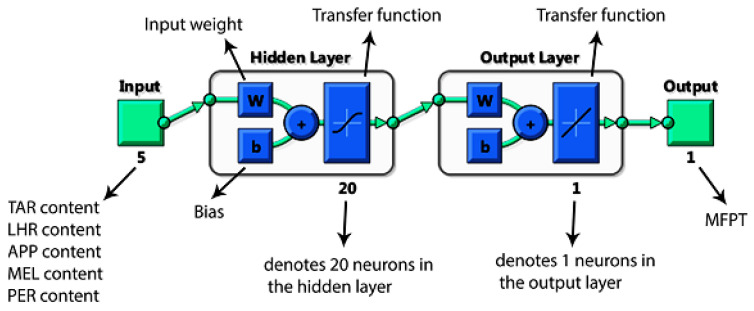
The schematic architecture of the ANN model that was used to predict the mean fireproofing time of the intumescent flame-retardant coating. Adapted with permission from ref. [99]. Copyright 2013 Fire Safety Journal.

A study carried out by Arabasadi et al. [99] was another instance that demonstrated the power of using AI to predict the properties of flame-retardant materials. A combination of AI techniques, including an ANN, an ANFIS, and a GA, was employed to predict the mean fireproofing time (MFPT) of intumescent coating on steel substrates. In this research, the components of the prepared intumescent coating were ammonium polyphosphate (APP), pentaerythritol (PER), melamine (MEL), thermoplastic acrylic resin (TAR), liquid hydrocarbon resin (LHR), and titanium dioxide (TiO_2_). They were considered to be five factors affecting the fire-retarding behavior of the intumescent coatings. APP, PER, and MEL act as the blowing agents, TAR acts as a binder, LHR as a plasticizer, and TiO2 acts as a pigment. Each of the components mentioned above was prepared with four different levels to form the resultant compounds. Based on Taguchi experimental design [100], 16 types of samples were manufactured to serve the heat insulation tests. Further details of 16 intumescent coating formulations with different factors and levels can be found in the paper [99]. For the construction of the AI-based model, APP, PER, MEL, TAR, and LHR acted as five independent input variables, while the MFPT was the output parameter of the system. The schematic architecture of the neural network applied in this study is provided in Figure 6.

**Figure 7:**
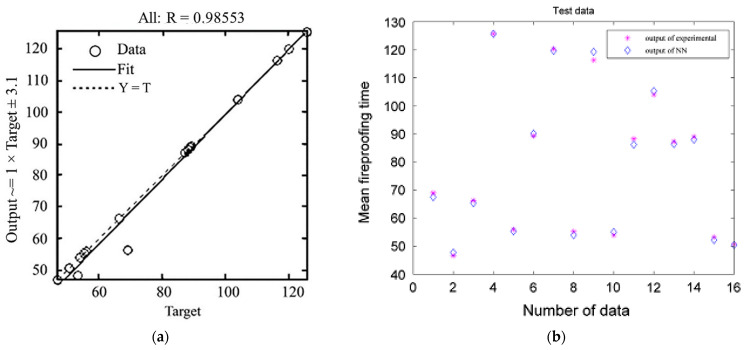
(**a**) The regression plot of the ANN-predicted result against the experimental data. (**b**) A comparison between the predicted outcomes of the ANN model and the practical results in terms of the mean fireproofing time (MFPT). Adapted with permission from ref. [99]. Copyright 2013 Fire Safety Journal.

For the training of the ANN model, 80%, 10%, and 10% of the original dataset were allocated for the training, testing, and validation sets, respectively. The statistical results of the ANN model in terms of MSE, RMSE, and R^2^ are summarized in Table 3. The RMSE values close to 0 and R^2^ values approaching 1 displayed that the ANN model was well-defined with the best fit between the predicted and measured output. A comparison between the predicted MFPT values from the ANN model and the actual experimental results is visualized in Figure 7. It can be clearly seen from the figure that most of the predicted results were in good agreement with the observed outcomes. However, some minor deviations between the predicted data and experimental outputs could be observed in Figure 7b, and they could be explained by some chemical- or physical-related phenomena. For example, the low concentration of the binder (TAR) compared to that of other constituents in the intumescent coating led to the removal of the coating layer from the steel surface, which minimized the fireproofing characteristic of the applied intumescent coating. Consequently, such phenomena resulted in lower values of MFPT than expected, and more importantly, less than what the ANN anticipated.

**Figure 8:**
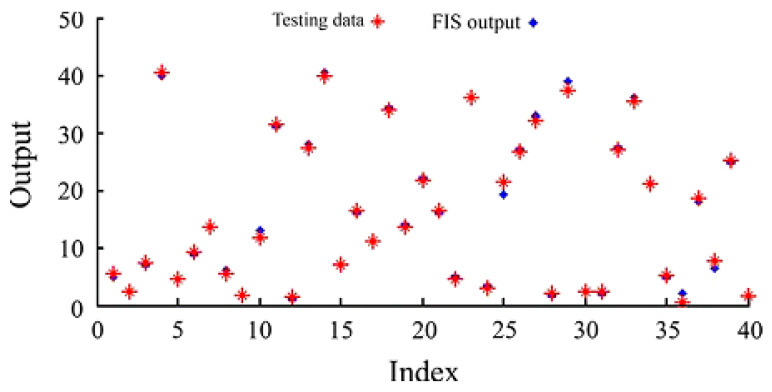
A comparison between the fuzzy inference system (FIS) outputs and testing data. Adapted with permission from ref. [99]. Copyright 2013 Fire Safety Journal.

The ANN model did not consider the nature of the relationship between input variables. In other words, the real interaction of the intumescent components in the coating was neglected by applying an ANN, which was the shortcoming of this approach. Therefore, the FIS appeared to the solution to overcome this inefficiency. By applying fuzzy rules in ANFIS modeling, the minimum contents of ATR and LHR constituents could be defined to prevent the foam detachment or char cracking phenomena from happening. Figure 8 shows a comparison between the FIS output and the testing data. The result obtained by the ANFIS model fitted the experimental data very well. Because the fireproofing properties of intumescent coatings primarily rely on experimental phenomena, and due to the absence of rule viewing, the use of only ANN modeling could generate outputs with unexpected errors. However, the utilization of the ANFIS model based on fuzzy rules was capable of obtaining a higher level of accuracy than that obtained when only applying an ANN.

Additionally, in this study, the GA, which was developed from Darwin’s natural selection approach, was used to find out the optimal formulation of the intumescent fireproofing coating. A comparison between the result obtained by the GA and the Taguchi analysis is shown in Table 4. It could be seen that a higher MFPT value was produced by the formulation by the GA modeling—about 2.79% higher than that of formulation obtained by the Taguchi experimental design. This minor difference proved the predictive capability of the GA in the optimization of intumescent coatings.

**Figure 9:**
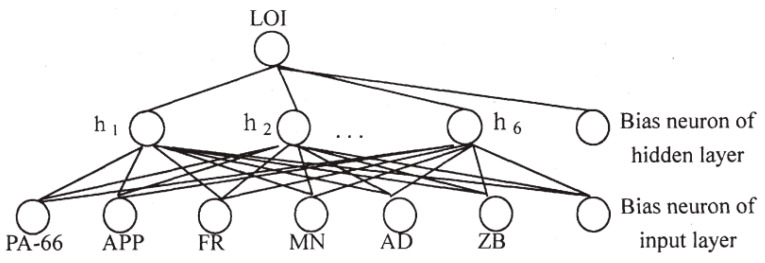
The schematic of the backpropagation network for the polyamide-66 (PA-66) formulation design model to predict the limiting oxygen index (LOI) value. Adapted with permission from ref. [101]. Copyright 2001 Journal of Fire Sciences.

In another piece of remarkable research by Xia et al. [101], the formulation design of halogen-free flame-retardant composites polyamide-66 (PA-66) was implemented by applying machine learning. The ANN was integrated into and ran on the platform of the Flame Retardant Expert System 2.0 (FRES 2.0) software. A three-layered backpropagation network, including six input nodes, six hidden nodes, and one output node, was employed in this study and is presented in Figure 9. A dataset with 30 samples corresponding to thirty formulations of polyamide-66 was used to train the machine learning model. Six independent constituents of composites consisting of PA-66, APP, phosphorus-containing flame retardant (FR), melamine (MN), silicon-containing additive (AD), and zinc borate (ZB) were considered as the input modeling variables. The limiting oxygen index (LOI) was targeted as the dependent output of the neural network.

**Figure 10:**
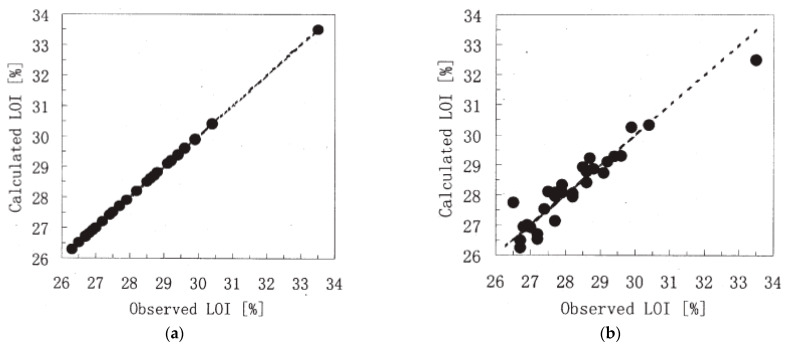
The correlation between the predicted and observed LOI values of (**a**) the MNLR model and (**b**) the ANN model. Adapted with permission from ref. [101]. Copyright 2001 Journal of Fire Sciences.

Another model constructed based on the multiple nonlinear regression (MNLR) analysis was also adopted in this study. A comparison between the results obtained by the MNLR and ANN models, as well as the predicted–observed LOI correlation, is illustrated in Figure 10. In terms of evaluation scores, the values of correlation coefficient (R) and RMSE given by the MNLR and ANN models are listed in Table 5. With an R-value of precisely 1.0 and an RMSE of nearly 0, the ANN model provided more accurate predictions compared to the MNLR model in terms of the LOI value of halogen-free, flame-retardant PA-66 compounds. In other words, the predicted LOI value of PA-66 composites was achieved with the aid of an ANN with a very high precision.

**Figure 11:**
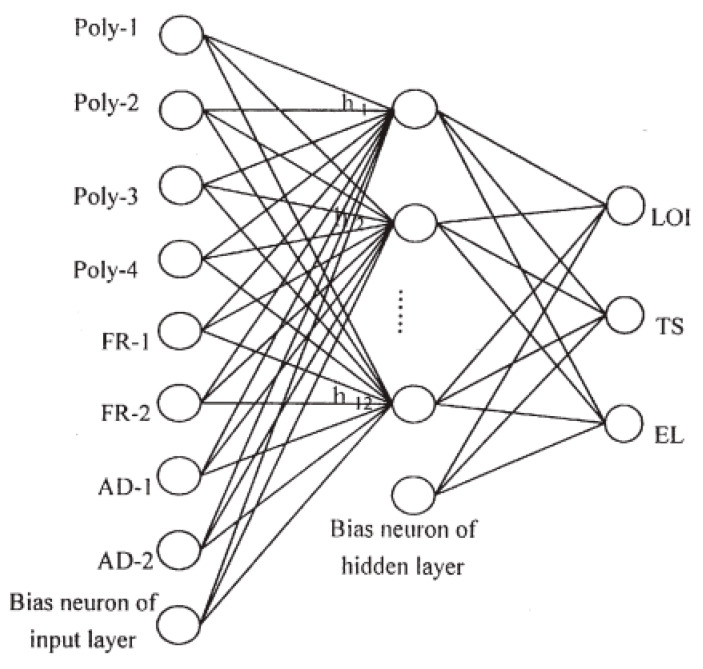
The schematic of the ANN to predict the LOI, tensile strength (TS), and elongation (EL) values of flame-retardant composites. Adapted with permission from ref. [102]. Copyright 2001 Journal of Fire Sciences.

Following the successful application of machine learning to predict the LOI value of polymers PA-66, Xia et al. continued to employ the FRES 2.0 software in another study in predicting tensile strength (TS) and elongation (EL) at break under mechanical testing [102]. The material prepared for this study was also halogen-free flame-retardant composites that was composed of ethylene-vinyl acetate copolymer (Poly-1), ethylene-propylene copolymer (Poly-2), polyethylene (Poly-3), a compatibilizer (Poly-4), alumina trihydrate (FR-1), zinc borate (FR-2), silicon-containing additive (AD-1), phosphorus-containing additive (AD-2), and antioxidant and processing agents. To simplify the ANN model, the concentration of antioxidant and processing agent was unchanged during the material preparation phase. Therefore, there were eight input parameters involved in the construction of the neural network, which were Poly-1, Poly-2, Poly-3, Poly-4, FR-1, FR-2, AD-1, and AD-2. Three target outputs of the modelling approach were LOI, TS, and EL. To train the machine learning model, a dataset of twenty-nine samples equivalent to twenty-nine different composite formulations was used. Among them, 20 out of 29 samples were used to train the ANN model, while the remaining nine sets were allocated to the test set to assess the predictability of the proposed model. Figure 11 illustrates the architecture of a three-layered backpropagation network applied in predicting LOI, TS, and EL values.

**Figure 12:**
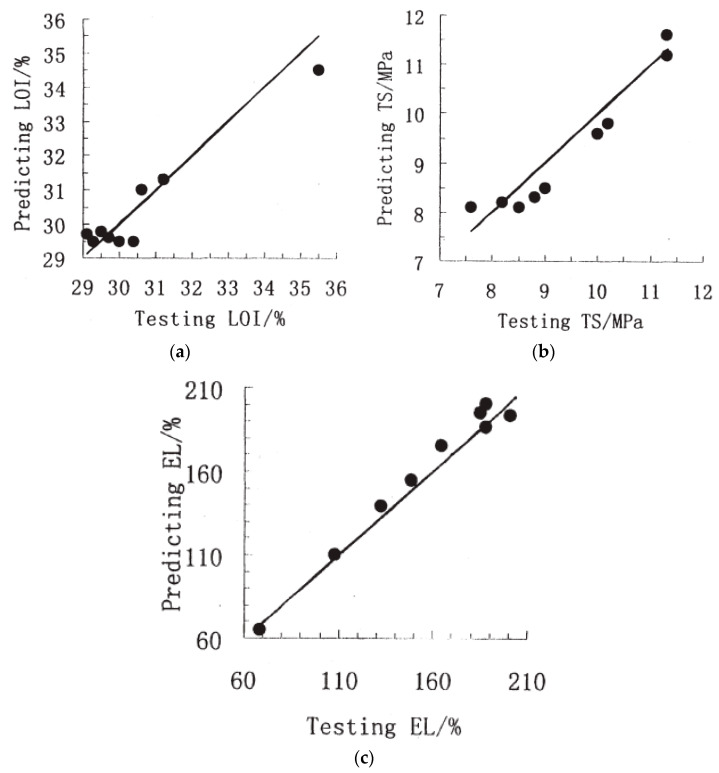
The correlation between the predicted and observed values of the (**a**) LOI, (**b**) TS, and (**c**) EL of the samples in the test set. Adapted with permission from ref. [102]. Copyright 2001 Journal of Fire Sciences.

The predictive power of the neural network in the determination of the LOI, TS, and EL values was objectively evaluated based on the test set. It is demonstrated in Table 6 through the terms of RMSE and R. It could be stated that the AI-based model proved its capability in the prediction of some characteristics of flame-retardant composites by providing the almost-one R values and relatively small RMSEs (close to 0). The correlation between the predicted and actual data for nine samples in the test set can be visualized in Figure 12, which shows the potential of applying the three-layered backpropagation network in fire predictions.

The typical descriptors applied in AI-based models of the above-mentioned studies to predict various characteristics of flame-retardant composites are summarized in Table 7. The importance of flame-retardants agents (i.e., boric acid, ammonium sulfate, ammonium polyphosphate, and alumina trihydrate) in the construction of the machine learning models was noticed. Without this type of descriptors, the machine learning model could not be properly built up and accurately predicted.

## 5. Advantages and Challenges

By reviewing the literature, the merit of utilizing machine learning models to evaluate the fire behavior of flame-retardant materials could be seen to be significant. Before AI techniques, it was a matter of time to understand the fire-related behavior of materials. To interpret this kind of phenomena, it mainly relied on the arrangement of the experimental program or development of a mathematical model based on reliable testing results. However, the main challenges that lowered the accuracy of these models were the required modelling resources with significant needed assumptions. After that, fine element modeling emerged as a helpful method to carry out a greater number of analyses. Nevertheless, it was necessary to undergo a time-consuming validation process with empirical parameters before the numerical models could be applied. However, unlike the methods mentioned above, the AI-based model enabled researches to carry out analyses with minimal human intervention. AI modeling not only helps to reduce calculation time a lot but also helps to solve unknown parameters that are not achievable through testing. Another exciting aspect of using AI modeling compared to advanced simulation is that increasing the number of data points is equivalent to reducing the mesh size (or increasing the number of mesh elements), which could result in significant improvement of model accuracy. It could, however, lead to a considerable increase in the computational time of advanced numerical models, which does not, on the other hand, occur in AI-based models. In other words, AI-model implementation was found to be quick, easy to use, and potentially affordable compared to other advanced simulation/calculation approaches.

However, the utilization of AI-/ML-based models is not entirely beneficial. Before training the model, this advanced approach requires a dataset with a sufficient number of data points because the predictive power or accuracy of a machine learning model is primarily dependent on the number of measured input data points. However, to gather enough data points for an ML model is not always a simple task. Some datasets might be built up based on the information collected from a literature review. However, if the literature cannot provide the needed information, the implementation of an experiment is essential. The number of available fire tests is still limited, which is also one of the challenges in the fire engineering discipline in general. Through an experimental test, the input variables of the model and the target output parameters can precisely be revealed. Therefore, while some advantages have been demonstrated over other conventional approaches, it is recommended that the advanced AI approach is better used to assess the fire-related behavior of materials in combination with traditional techniques such as experimental testing or numerical simulation.

## 6. Recommendation for Future Studies

From the perspective of fire engineering, the limitation of the number of available fire tests hinders the development of models based on AI/ML. To address this challenge, the research community should work together to synthesize the findings of previous fire studies and set up new fire tests to generate the new fire data. This would not only help to better understand the fire-induced behavior of the materials but also allow for the creation of more complete datasets that serve the purpose of building AI/ML models. Consequently, the performance of AI/ML-based models could be significantly improved once more data points become available. Moreover, one of the drawbacks of AI modeling is that this approach does not consider the nature of the relationship between input and output variables, which can only be better revealed by performing fire experiments. Therefore, to get a comprehensive understanding of the behavior of materials under fire conditions, the use of AI/ML-based models in conjunction with experimental studies in compliance with regulations and standards should be performed. Another solution to tackle the shortage of databases is constructing validated finite element models to generate the raw input data points required for AI models [89]. This approach will primarily rely on the results of fire tests to build up validated finite element models.

## 7. Conclusions

This review paper has provided a comprehensive look at the use of artificial intelligence in evaluating the behavior of construction materials, a variety of structural elements/systems, and their flame-retardant capabilities under fire scenarios. The detailed schematic architecture of neural networks and encouraging statistical results were given in evaluating the fire performance of flame-retardant materials, which proves the powerful capability of AI/ML-based models in this area. Through this review, some advantages and challenges in applying AI techniques compared to conventional methods (e.g., experimental tests and advanced simulations) in the fire engineering area were noticed, and constructive recommendations for future researches were given. This review paper is expected to offer benefits to scientists in the fields of fire engineering and material science, as well as to enable them to explore and consider the use of AI/ML in their research projects.

Through this review, some remarks could be drawn as follows:

- AI techniques have been extensively applied to evaluate the fire performance of different construction materials consisting of concrete, steel, timber, and composites, with encouraging results. AI-based models have also shown the potential to predict the behavior of a variety of structural components such as beams, columns, slabs, frames, trusses, and connections under fire scenarios.
- Some ML and AI algorithms have commonly been used in the evaluation of the behavior of materials/structural systems exposed to fires, including ANNs, the ANFIS, and the GA. While neural networks have mostly been applied to simulate the nonlinear relationship between various input descriptors and the target output, the GA has been employed to generate the required input parameters for the computational approach.
- ML techniques have brought many advantages compared to conventional approaches, such as saving computing time, providing a high level of accuracy, and implementing with minimal human intervention. However, some drawbacks of these advanced techniques could be noticed, such as requiring database construction with adequate data points for ML-based models or being unable to simulate the essence of the input–output relationship for fire engineering problems.
- For the purpose of constructing AI/ML models, it is suggested that further fire tests should be arranged to generate the fire database. Additionally, reliable finite element models could be constructed and validated to provide additional input data points to be used in AI models. The AI approach should work in conjunction with traditional methods (e.g., experimental tests and numerical simulations) to better understand the fire phenomena and flame retardancy of construction materials.

## Figures and Tables

**Table 2:** The statistical metrics of the ANN model used to predict the modulus of rupture (MOR) and mass loss (MLoss) of fiberboard [98]. RMSE: root mean square error; R^2^: coefficient of determination.

Data	R^2^		RMSE	
	MLoss	MOR	MLoss	MOR
All datasets	0.91882	0.92117	0.1725	1.3033
The training set	0.96919	0.95721	0.1545	1.2025
The validation set	0.99899	0.92552	1.4846	1.9196
The test set	0.93291	0.96552	0.0836	1.9914

**Table 3:** The statistical metrics of the ANN model used to predict the mean fireproofing time of the intumescent flame-retardant coating [99].

Procedure	R^2^	RMSE	MSE
Training	1	0.01229	0.01512
Validating	0.96120	0.02519	0.06345
Testing	0.99562	0.01954	0.03818
All data	0.98553	-	-

**Table 4:** A comparison between Taguchi and the genetic algorithm (GA) methods in terms of the optimal intumescent coating formulation and the MFPT value [99]. APP: ammonium polyphosphate; PER: pentaerythritol; MEL: melamine; TAR: thermoplastic acrylic resin; and LHR: liquid hydrocarbon resin.

Methods	TAR (g)	LHR (g)	APP (g)	MEL (g)	PER (g)	MFPT (Min)
Taguchi	13	2.5	25	10	11	129.2
GA	15.85	2.8	26.7	10.01	8.4	132.8

**Table 5:** A comparison of prediction capability between multiple nonlinear regression (MNLR) analysis and the ANN model [101].

Methods	Correlation Coefficient	Root-Mean-Square Error
MNLR model	0.9474	0.4388
ANN model	1.0000	0.0002

**Table 6:** The statistical results of the ANN model for the samples in the test set [102].

Output Results	Correlation Coefficient	Root-Mean-Square Error
LOI (%)	0.9524	0.38
TS (MPa)	0.9557	0.54
EL (%)	0.9695	10.10

**Table 7:** Typical descriptors used in AI/ML models to predict the fire performance of flame-retardant materials.

Materials	Method	Target Output	Descriptors/Input Parameters	Reference
Flame-retardant fiberboards	Artificial neural networks	The modulus of rupture (MOR) and mass loss (ML)	The concentration of boric acid, borax, and ammonium sulfate, and press temperature	[98]
Intumescent flame-retardant coatings	Artificial neural networks (ANNs), adaptive neuro-fuzzy-inference-system (ANFIS), and genetic algorithm (GA)	The mean fireproofing time (MFPT)	The compositional concentration of ammonium polyphosphate (APP), pentaerythritol (PER), melamine (MEL), thermoplastic acrylic resin (TAR), and liquid hydrocarbon resin (LHR)	[99]
Polyamide-66	Artificial neural networks	The limiting oxygen index (LOI)	Constituents of composites: polyamide-66 (PA-66), ammonium polyphosphate (APP), phosphorus-containing flame retardant (FR), melamine (MN), silicon-containing additive (AD), and zinc borate (ZB)	[101]
Halogen-free flame-retardant composites	Artificial neural networks	The limiting oxygen index (LOI), tensile strength (TS), and elongation (EL)	Ethylene-vinyl acetate copolymer (Poly-1), ethylene-propylene copolymer (Poly-2), polyethylene (Poly-3), compatibilizer (Poly-4), alumina trihydrate (FR-1), zinc borate (FR-2), silicon-containing additive (AD-1), and phosphorus-containing additive (AD-2)	[102]

## Data Availability

Not applicable.
